# Body mass index, abdominal fatness, and the risk of sudden cardiac death: a systematic review and dose–response meta-analysis of prospective studies

**DOI:** 10.1007/s10654-017-0353-9

**Published:** 2018-02-07

**Authors:** Dagfinn Aune, Sabrina Schlesinger, Teresa Norat, Elio Riboli

**Affiliations:** 1 Department of Epidemiology and Biostatistics, School of Public Health, Imperial College London, St. Mary’s Campus, Norfolk Place, Paddington, London, W2 1PG UK; 2 Bjørknes University College, Oslo, Norway; 3 Department of Endocrinology, Morbid Obesity and Preventive Medicine, Oslo University Hospital, Oslo, Norway; 4 Institute for Biometrics and Epidemiology, German Diabetes Center (DDZ), Heinrich Heine University Düsseldorf, Düsseldorf, Germany

**Keywords:** Obesity, BMI, Waist-to-hip ratio, Sudden cardiac death, Meta-analysis

## Abstract

Although overweight and obesity are established risk factors for some types of heart disease including ischemic heart disease, heart failure and atrial fibrillation, less is known about the association between adiposity and sudden cardiac death. We conducted a systematic review and meta-analysis of prospective studies to clarify the association between adiposity and risk of sudden cardiac death. PubMed and Embase databases were searched up to July 20th 2017. Summary relative risks (RRs) and 95% confidence intervals (CIs) were calculated using random effects models. The summary RR was 1.16 (95% CI 1.05–1.28, I^2^ = 68%, n = 14) per 5 unit increment in BMI, and 1.82 (95% CI 1.61–2.07, I^2^ = 0%, n = 3) per 0.1 unit increase in waist-to-hip ratio, and 1.03 (95% CI 0.93–1.15, I^2^ = 0%, n = 2) per 10 cm increase in waist circumference. The heterogeneity in the analysis of BMI and sudden cardiac death persisted across most subgroup analyses. The association was stronger among studies with longer follow-up compared to short follow-up and was observed in the European and American studies, but not in the Asian studies. There was a J-shaped association between BMI and sudden cardiac death and the lowest risk was observed in the normal weight range, however, the increased risk with a low BMI was attenuated among studies with a longer duration of follow-up. This meta-analysis suggest an increased risk of sudden cardiac death with increasing BMI and waist-to-hip ratio, however, further studies with stratification for smoking status are needed of waist circumference, weight changes and adiposity at younger ages.

**Electronic supplementary material:**

The online version of this article (10.1007/s10654-017-0353-9) contains supplementary material, which is available to authorized users.

## Introduction

The prevalence of overweight and obesity has increased rapidly over the last decades in all areas of the world [1]. Overweight and obesity are important risk factors for a wide range of chronic diseases, including cardiovascular diseases, type 2 diabetes, several types of cancer as well as all-cause mortality [2–10], and the current trends are a major challenge for public health both in terms of reduced quality of life and increased medical costs [11].

Sudden cardiac death accounted for more than 350,000 deaths in the USA in 2014 [12]. Although clinical guidelines focus on reducing risk in high-risk patients using medical therapies, up to 75% of all sudden cardiac deaths occur in patients not classified as high-risk by current guidelines [13]. Although a substantial amount of data has consistently showed that overweight and obesity increases the risk of coronary heart disease [5], heart failure [6] and atrial fibrillation [14], all risk factors for sudden cardiac death, data are more limited and less consistent regarding the association between overweight and obesity and the risk of sudden cardiac death [15–27]. Some studies showed an increase in risk of sudden cardiac death with a greater body mass index (BMI, weight in kg divided by height squared in metres, kg/m^2^) [16, 25, 27], however, other studies found associations only in non-current smokers [26], no significant association [15, 20, 21], or even some increase in risk with a low BMI [18]. Given the increased prevalence of overweight and obesity globally establishing whether excess BMI also is related to increased risk of sudden cardiac death is of major public health importance and could inform guidelines for prevention. For these reasons we conducted a systematic review and meta-analysis of prospective studies of adiposity and the risk of sudden cardiac death. We aimed to clarify the direction and strength of the association, shape of the dose–response relationship and potential sources of heterogeneity between studies.

## Methods

### Search strategy and inclusion criteria

We searched the PubMed and Embase databases up to July 20th 2017 for eligible studies (DA, and SS). A list of the search terms used are provided in Supplementary Table 1 and 2. We followed standard criteria for reporting of meta-analyses [28]. In addition, we searched the reference lists of the relevant publications for further studies. Study quality was assessed using the Newcastle–Ottawa scale [29].

### Study selection

We included prospective and retrospective cohort studies and nested case–control studies of the association between adiposity measures (BMI, waist circumference, waist-to-hip ratio, hip circumference, and weight gain) and risk of sudden cardiac death that were published in English. Studies in high-risk populations (patient populations), abstract only publications, grey literature and unpublished studies were excluded. Adjusted relative risk estimates (hazard ratio, risk ratio, or odds ratio) had to be available with the 95% confidence intervals (CIs) in the publication and for the dose–response analysis, a quantitative measure of adiposity and the total number of cases and person-years or non-cases had to be available in the publication. When multiple publications were available from the same study we used the study with the largest number of cases. A list of the excluded studies and exclusion reasons are found in the Supplementary Table 3.

### Data extraction

We extracted the following data from each study: The first author’s last name, publication year, country where the study was conducted, study period, sample size, number of cases/controls, exposure variable, exposure level, relative risks and 95% confidence intervals for the highest versus the lowest level of the exposure variable and variables adjusted for in the analysis. Data were extracted by one reviewer (DA) and checked for accuracy by a second reviewer (SS). Any disagreements were resolved through discussion.

### Statistical analysis

We calculated summary RRs and 95% CIs for a 5 unit increment in BMI, a 0.1 unit increment in waist-to-hip ratio, and 10 cm increase in waist circumference (approximately equal to one standard deviation for each measure) using a random effects model, which takes into account heterogeneity between studies [30]. For the primary analysis we used the model from each study that had the greatest degree of control for potential confounding with the exception of when potential intermediate risk factors were adjusted for in a separate step (as an exploration of how much of the association might be mediated by cholesterol for example). The average of the natural logarithm of the RRs was estimated and the RR from each study was weighted according to the method of DerSimonian and Laird [30]. A two-tailed *p* < 0.05 was considered statistically significant. If studies reported results separately for men and women or other subgroups we combined the subgroup-specific estimates using a fixed-effects model to generate an overall estimate so that each study was only represented once in the main analysis, but sex-specific results are presented separately in subgroup analyses.

The method described by Greenland and Longnecker [31] was used for the dose–response analysis of adiposity measures and we calculated study-specific slopes (linear trends) and 95% CIs from the natural logs of the reported RRs and CIs across categories of each adiposity measures. The mean level of BMI or WHR in each category was assigned to the corresponding relative risk for each study and for studies that reported the exposures in ranges we calculated the average of the upper and the lower cut-off point which was used as a midpoint. When the lowest or highest category was open-ended or had an extreme range we used the width of the adjacent interval to calculate an upper or lower cut-off value. For studies that reported continuous risk estimates per 1 BMI unit or per 3.3 BMI units these risk estimates were converted to a risk estimate per 5 BMI units by first taking the natural logarithm of the RR (95% CI), then dividing the ln(RR, 95% CI) by the increment reported, then multiplying by 5, and back-transforming to non-logarithmic scale before inclusion in the meta-analysis. A potential nonlinear dose–response relationship between BMI, waist-to-hip ratio, and waist circumference and risk of sudden cardiac death was examined by using fractional polynomial models [32]. We determined the best fitting second order fractional polynomial regression model, defined as the one with the lowest deviance. A likelihood ratio test was used to assess the difference between the nonlinear and linear models to test for nonlinearity [32]. Studies that only reported a continuous risk estimate and not categorical data were excluded in the nonlinear dose–response analysis as it requires that data are reported for at least three categories of BMI.

Subgroup analyses stratified by sex, measurement versus self-report of adiposity measures, duration of follow-up, geographic location, number of cases, study quality scores, and adjustment for confounders (age, smoking, alcohol, physical activity) and potential intermediates (hypertension, blood pressure, cholesterol, diabetes mellitus, coronary heart disease, heart failure, and left ventricular hypertrophy) were conducted to investigate potential sources of heterogeneity and heterogeneity between studies was quantitatively assessed by the Q test and I^2^ [33]. Meta-regression analyses were used to examine between subgroup differences in the summary estimates. Small study effects, such as publication bias, were assessed by inspecting the funnel plots for asymmetry and with Egger’s test [34] and Begg’s test [35] with the results considered to indicate small study effects when *p* < 0.10. Sensitivity analyses excluding one study at a time were conducted to clarify whether the results were simply due to one large study or a study with an extreme result. The statistical analyses were conducted using Stata software version 13.0 (StataCorp, College Station, TX, USA).

## Results

**Fig. 1:**
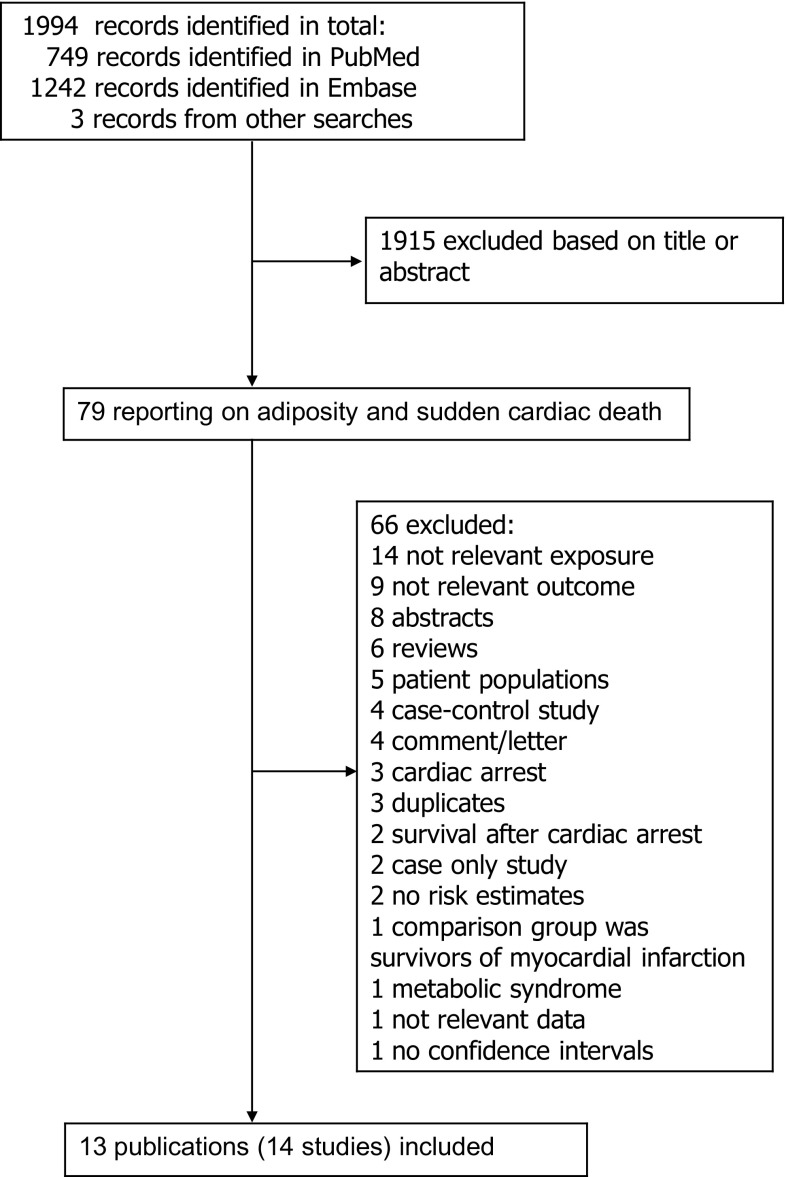
Flow-chart of study selection

We identified 14 prospective studies (13 publications) [15–27] that were included in the systematic review of BMI, waist-to-hip ratio, and waist circumference and risk of sudden cardiac death (Supplementary Table 4; Fig. 1). Only one study reported on BMI in early adulthood (at age 18 years) [25] or weight change [25] and sudden cardiac death and meta-analyses were therefore not possible for these measures. All studies reported on BMI at baseline and the studies were conducted mostly in middle-aged populations. The age range or mean age for each study is provided in Supplementary Table 4 and the lower and higher age range across studies was 30 and 84 years, respectively. The mean (median) duration of follow-up was 16.4 (11.8) years and ranged from 5.2 to 38 years. Three studies included only men, two included only women and nine studies included both men and women. Eight studies were from Europe, three were from the USA, and three were from Asia (Japan) (Supplementary Table 4).

### Body mass index

**Fig. 2:**
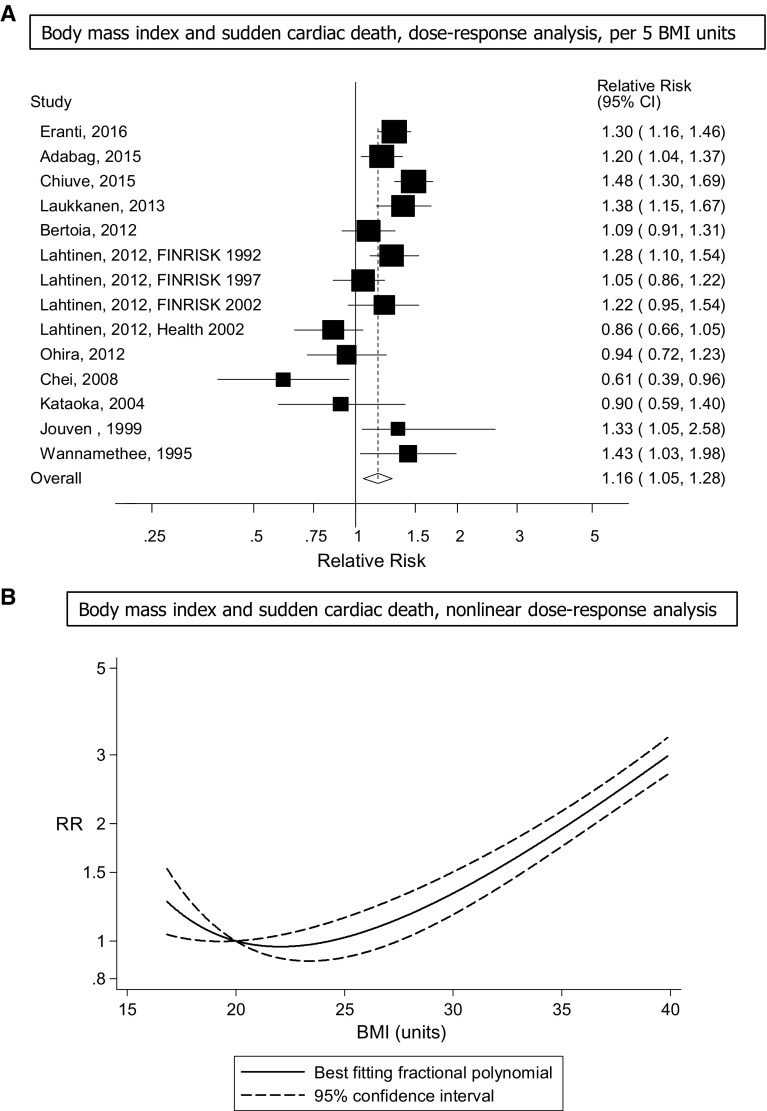
BMI and sudden cardiac death, linear and nonlinear dose–response analysis

Fourteen prospective studies (11 publications) [15–18, 20–22, 24–27] were included in the linear dose–response analysis of BMI and sudden cardiac death incidence and included 3376 cases among 406,079 participants. The summary RR for a 5 unit increment in BMI was 1.16 (95% CI 1.05–1.28, I^2^ = 68.2%, p_heterogeneity_ < 0.0001) (Fig. 2a). All but one of the studies found increased risk, but the strength of the association differed between studies. In sensitivity analyses excluding the most influential studies, the summary RR ranged from 1.14 (95% CI 1.03–1.27) when excluding the Nurses’ Health Study [25] to 1.20 (95% CI 1.09–1.31) when excluding the Health 2002 study [22] (Supplementary Table 5). There was no indication of publication bias with Egger’s test, *p* = 0.18, or with Begg’s test, *p* = 0.27, and there was no evidence of asymmetry by inspection of the funnel plot (Supplementary Figure 1). Seven studies [15, 18, 20, 21, 25–27] were included in the nonlinear dose–response analysis. There was evidence of a nonlinear J-shaped association between BMI and sudden cardiac death, p_nonlinearity_ < 0.0001 (Fig. 2b, Supplementary Table 6) with a slight increase in risk in the underweight categories, and a 14%, 60% and 2–3 fold increase in risk in the overweight, obese, and severely obese categories. When the nonlinear dose–response analysis was stratified by duration of follow-up the association between low BMI and increased risk of sudden cardiac death was stronger among the studies with < 10 years follow-up than among studies with ≥ 10 years follow-up, and in addition, the optimal BMI was around 23 and 20–22 among the studies with short and long-follow-up duration, respectively (Supplementary Figures 2, 3).

### Waist-to-hip ratio and waist circumference

**Fig. 3:**
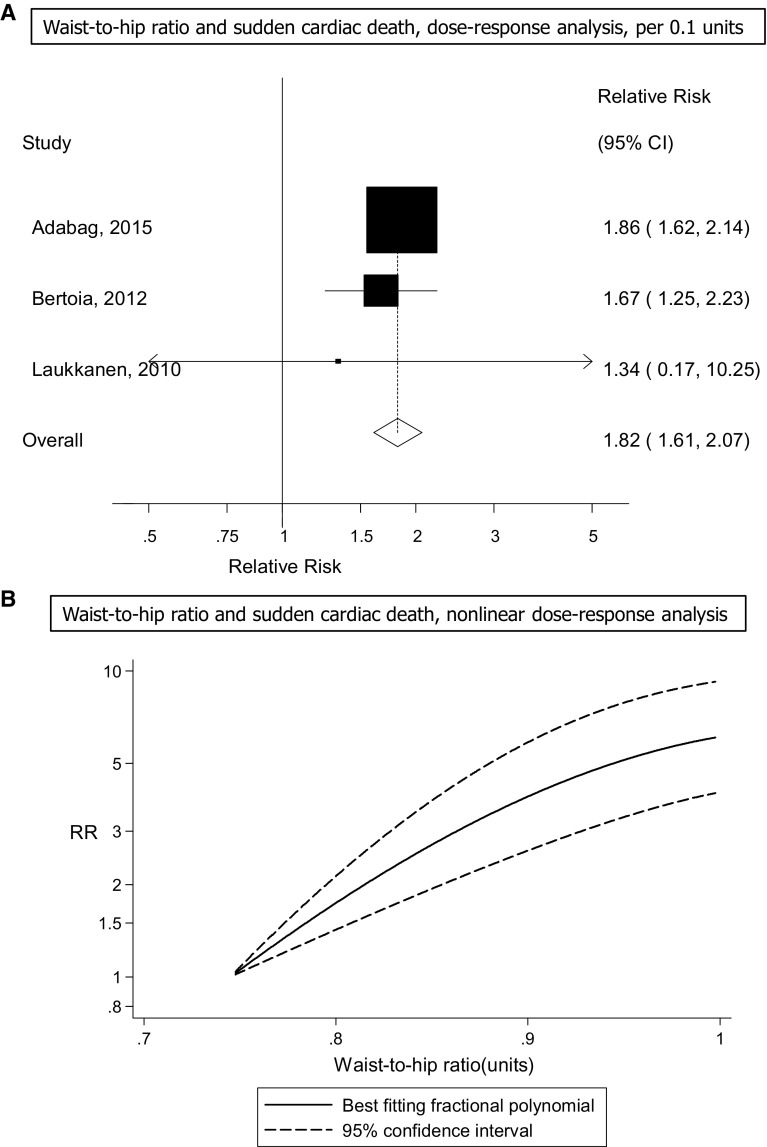
Waist-to-hip ratio and sudden cardiac death

Three prospective studies [19, 21, 26] were included in the analysis of waist-to-hip ratio and risk of sudden cardiac death (817 cases, 179,117 participants) and the summary RR for a 0.1 unit increment in waist-to-hip ratio was 1.82 (95% CI 1.61–2.07, I^2^ = 0%, p_heterogeneity_ = 0.77) (Fig. 3a). Although the test for nonlinearity was significant, p_nonlinearity_ = 0.02, there was no evidence of a threshold effect and the association increased strongly even with modest increases in waist-to-hip ratio (Fig. 3b, Supplementary Table 7).

**Fig. 4:**
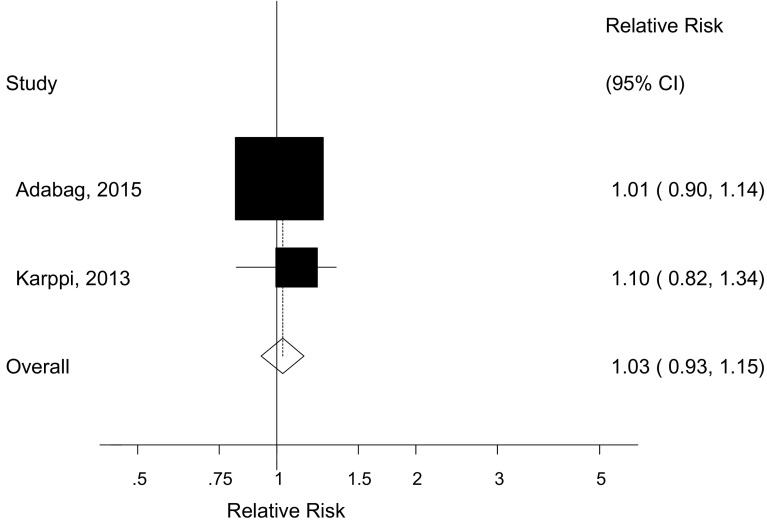
Waist circumference and sudden cardiac death, per 10 cm

Two prospective studies [23, 26] reported on waist circumference and sudden cardiac death (312 cases, 15,972 participants) and the summary RR for a 10 cm increase was 1.03 (95% CI 0.93–1.15, I^2^ = 0%, p_heterogeneity_ = 0.54) (Fig. 4). Nonlinear dose–response analysis was not possible because only one of the studies reported categorical data.

### Subgroup and sensitivity analyses and study quality

In subgroup analyses of the association between BMI and sudden cardiac death there was suggestive heterogeneity when the analyses were stratified by duration of follow-up, p_heterogeneity_ = 0.06, with stronger associations among studies with longer duration of follow-up, and when analyses were stratified by geographic location, p_heterogeneity_ = 0.09, with no association among the Asian studies, while a positive association was observed among the European and North American studies (Table 1). There was no significant heterogeneity between the remaining subgroup analyses and there were positive associations in most of them although the association was not always significant possibly because of the limited number of studies in some subgroups. Study quality was high with a mean (median) score of 7.6 [8] out of 9 points (Supplementary Table 8).

**Table 1:** Subgroup analyses of BMI and sudden cardiac death

	BMI				
	*n*	RR (95% CI)	I^2^ (%)	*P*_h_^a^	*P*_h_^b^
All studies	14	1.16 (1.05–1.28)	68.2	< 0.0001	
Sex					
Men	3	1.11 (0.70–1.75)	70.7	0.03	0.82/0.99^c^
Women	3	1.16 (0.83–1.62)	82.8	0.003	
Men and women	9	1.15 (1.04–1.27)	59.2	0.01	
Assessment of weight/height					
Measured	10	1.19 (1.10–1.30)	47.8	0.05	0.21
Self-reported (validated)	2	0.98 (0.41–2.33)	92.8	< 0.0001	
Not available	2	0.93 (0.74–1.17)	0	0.88	
Duration of follow-up					
< 5 years	0				0.06
5–< 10 years	5	0.99 (0.76–1.28)	70.6	0.009	
10–< 15 years	3	1.12 (1.02–1.23)	0	0.49	
15–< 20 years	2	1.32 (1.17–1.50)	0	0.54	
≥ 20 years	4	1.28 (1.08–1.51)	67.8	0.03	
Geographic location					
Europe	8	1.21 (1.08–1.35)	55.8	0.03	0.09
America	3	1.26 (1.05–1.50)	77.7	0.01	
Asia	3	0.84 (0.66–1.07)	26.3	0.26	
Number of cases					
Cases < 100	2	0.89 (0.45–1.73)	85.8	0.008	0.44
Cases 100–< 200	6	1.18 (1.01–1.39)	64.3	0.02	
Cases ≥ 200	6	1.20 (1.05–1.36)	69.4	0.006	
Study quality					
0–3	0				0.35
4–6	1	0.94 (0.72–1.23)			
7–9	13	1.18 (1.07–1.30)	67.7	< 0.0001	
Adjustment for confounders					
Age					
Yes	14	1.16 (1.05–1.28)	68.2	< 0.0001	NC
No	0				
Smoking					
Yes	12	1.14 (1.02–1.28)	72.4	< 0.0001	0.49
No	2	1.23 (1.08–1.40)	0	0.32	
Alcohol					
Yes	4	1.11 (0.82–1.48)	85.6	< 0.0001	0.99
No	10	1.16 (1.07–1.27)	45.7	0.06	
Physical activity					
Yes	6	1.09 (0.89–1.34)	83.2	< 0.0001	0.54
No	8	1.21 (1.10–1.33)	36.7	0.14	
Adjustment for potential intermediates					
Hypertension					
Yes	4	1.03 (0.76–1.40)	87.0	< 0.0001	0.48
No	10	1.19 (1.09–1.31)	49.3	0.04	
Blood pressure					
Yes	8	1.17 (1.05–1.32)	58.1	0.02	0.89
No	6	1.13 (0.94–1.37)	79.2	< 0.0001	
Cholesterol					
Yes	6	1.27 (1.11–1.45)	60.1	0.03	0.24
No	8	1.10 (0.97–1.24)	62.0	0.01	
Diabetes mellitus					
Yes	13	1.11 (0.98–1.26)	73.6	< 0.0001	0.24
No	3	1.28 (1.15–1.42)	0	0.37	
Coronary heart disease					
Yes	7	1.17 (1.05–1.30)	62.4	0.01	0.90
No	7	1.13 (0.93–1.37)	75.2	< 0.0001	

*n* denotes the number of studies. The number of studies is not always equal to the total because the subgroup analyses were not applicable to some studies or information was not provided in the publication

^a^P for heterogeneity within each subgroup

^b^P for heterogeneity between subgroups

^c^P for heterogeneity between men and women (excluding men/women combined)

## Discussion

This is to our knowledge is the first meta-analysis of adiposity and the risk of sudden cardiac death. There was a 16% increase in the relative risk per 5 units increase in BMI and an 82% increase in relative risk per 0.1 unit increase in waist-to-hip ratio, based on eight and three studies, respectively, however, no association was observed among two studies of waist circumference. There was evidence of a nonlinear J-shaped association between BMI and sudden cardiac death, with a slight increase in risk in the underweight categories, and a 14%, 60% and 2–3 fold increase in risk in the overweight, obese and severely obese categories. The association between waist-to-hip ratio and sudden cardiac death was also nonlinear, but the dose–response curve was slightly steeper at lower levels of waist-to-hip ratio than at higher levels. Our current findings are consistent with previous studies that have found that adiposity increases the risk of other heart conditions that increase the risk of sudden cardiac death including coronary heart disease [5], heart failure [6], and atrial fibrillation [14].

Our meta-analysis has some limitations that need to be mentioned. Confounding by other risk factors may have influenced the results. We conducted several subgroup analyses to try to clarify whether adjustment for specific confounding factors influenced the summary estimates. The association between BMI and sudden cardiac death was in the direction of increased risk in most subgroup analyses, however, it was not significant in every subgroup analysis (possibly because there were few studies and low statistical power in some subgroups). More importantly, there was no heterogeneity between the subgroups analyses stratified by adjustment for confounding factors and potential intermediate factors. The association was not significant among the studies that adjusted for hypertension (which could be considered an intermediate factor), however, the association was significant among the studies that adjusted for blood pressure. The reason for the difference might be a mix of chance and limited statistical power in the analysis with adjustment for hypertension as there were only four studies in that subgroup analysis, while there were eight studies in the subgroup analysis with adjustment for blood pressure. The association persisted among studies that adjusted for serum cholesterol and coronary heart disease, but not among studies that adjusted for diabetes mellitus, but again there was no heterogeneity between the subgroups.

There was suggestion of a stronger association between BMI and sudden cardiac death among studies with longer compared to shorter durations of follow-up. There was no association among the studies with 5–< 10 years of follow-up, but a 12% increase in the relative risk per 5 BMI units among studies with 10 < 15 years follow-up and a 32% and 28% increase in the relative risk among studies with 15–< 20 and ≥ 20 years follow-up, respectively. The increased risk observed with a low BMI in the nonlinear dose–response analysis was also substantially attenuated among studies with longer (≥ 10 years) follow-up, while it was stronger among the studies with shorter (< 10 years) follow-up and the optimal BMI was around 23 among the studies with short follow-up, and around 20–22 among the studies with longer follow-up. This is similar to what we have previously found in relation to heart failure [6] and all-cause mortality [4] and might to some degree reflect confounding by illness and associated weight loss which may have a greater impact in studies with a short duration of follow-up than among studies with a long duration of follow-up [4, 6], or alternatively it might reflect weight gain over time which could contribute to increased risk beyond that of baseline BMI [25]. For example, if only a baseline anthropometric assessment was conducted in the included studies, people who were normal weight at baseline might become overweight, and people who were overweight might become obese (given trends with increased weight gain in most populations over time [1]), but because of only the baseline assessment they would still be categorized as normal and overweight, respectively. The studies with the longest duration of follow-up would then also be the studies were the weight increased the most over time and if weight gain increases risk of sudden cardiac death, those studies would show the strongest associations. Lastly, smoking may be a strong confounding factor of the association between BMI and health outcomes because smoking is strongly associated with a number of chronic diseases and mortality [36], but at the same time is associated with lower BMI [37] and may therefore confound and drive the optimal BMI upwards as observed in our previous analysis of BMI and all-cause mortality [4] and might also at least partly explain the observed slight increase in risk of sudden cardiac death with a low BMI of 16.7–17.5 compared to a BMI of around 20–22. Unfortunately it was not possible to conduct similar stratified analyses by smoking status in the current meta-analysis because only one of the included studies reported such stratified analyses [26]. However, that study reported RRs (95% CIs) of 1.73 (1.06–2.84), 1.94 (1.12–3.33), and 3.36 (1.86–6.07) for BMI categories of 25–29.9, 30–34.9, ≥ 35.0 compared to 18.5–24.9 among non-smokers and 1.39 (0.43–4.50), 0.86 (0.55–1.33, 0.94 (0.53–1.65), 0.34 (0.08–1.41) for BMI categories of < 18.5, and 25–29.9, 30–34.9, ≥ 35.0 compared to 18.5–24.9 among current smokers, strongly supporting a negative confounding effect of smoking on the association between BMI and sudden cardiac death.

Measurements of weight, height, waist and hip circumferences may have been affected by measurement errors, however, the association for BMI was significant only among the studies that used measured weight and height, not among those that used self-reported weight and height or those where the assessment of weight and height was unclear. This is probably also a chance finding as there was only two studies with either self-reported or unclear anthropometric measurements and because there was no heterogeneity between the different measurements of anthropometric factors. Validation studies have reported high correlations between self-reported and measured anthropometric measures [38–41]. BMI is an imperfect measure of body fatness as it does not distinguish between body fat and muscle mass. However, studies have shown high correlations between BMI and waist measures and body fat as measured by dual-energy x-ray absorptiometry (DXA) [42, 43]. The association between adiposity and sudden cardiac death was positive for both BMI and waist-to-hip ratio, although the dose–response relationship appeared to be stronger for the latter. Although publication bias or small study bias can affect the findings of meta-analyses of published literature, we found no evidence of such bias with Egger’s or Begg’s test. Because there was only one study on BMI in early adulthood and weight changes in relation to sudden cardiac death and because of the limited number of studies on waist circumference further studies are needed of these adiposity measures in relation to risk of sudden cardiac death.

Several mechanisms could explain an association between body fatness and increased risk of sudden cardiac death. Adiposity is associated metabolic disturbances including higher levels of total and LDL-cholesterol and lower HDL cholesterol [3], dyslipidemia [44], insulin resistance and diabetes [45] and inflammation which contributes to increased risk of sudden cardiac death [16, 46, 47]. Adiposity increases hemodynamic stress which activates the renin-antiotensin-aldosterone system leading to elevated aldosterone levels, which again increases blood volume and cardiac output and thereby contributes to left ventricular hypertrophy [48–51]. Adiposity also contributes to increased risk of hypertension [2] and higher resting heart rate [52, 53], which increases the risk of sudden cardiac death [22, 54]. Echocardiographic studies have shown that the heart adapts to obesity through eccentric cardiac hypertrophy, but to hypertension through concentric hypertrophy, and when both obesity and hypertension coexists, features of both concentric and eccentric ventricular hypertrophy results [55]. Obesity is associated with the secretion of cytokines that influence the heart’s physiology and structure. High levels of leptin observed in obesity increases myocardial fat, reduces contractility and hypertrophy [56]. Adiposity is associated with low-grade inflammation and elevated levels of inflammatory markers such as tumor necrosis factor-alpha which reduces adiponectin and thereby increases left ventricular hypertrophy through AMP kinase signaling and alpha-adrenergic receptor stimulation [57]. Insulin resistance contributes to left ventricular hypertrophy through IGF-1 receptor stimulation [58]. Obesity is also associated with increased risk of cardiomyopathy [59], which increases the susceptibility to ventricular arrhytmia and sudden cardiac death. Excess weight is also associated with increased risk of medical conditions such as coronary heart disease [60], heart failure [6], and atrial fibrillation [14], which are established risk factors for sudden cardiac death [16, 46, 47, 61]. Obese patients without and with eccentric left ventricular hypertrophy have been shown to have 10-fold and 30-fold increased risk of premature ventricular contractions, respectively, compared to lean persons [62]. Ventricular ectopy (three or more premature beats per hour) or ventricular tachycardia are associated with increased risk of mortality among patients with coronary artery disease, valvular disease and cardiomyopathy and has been shown to be responsible for three out of four ventricular fibrillation episodes [63, 64]. Necropsy studies have found epicardial fat infiltration of the sinus node, fatty and fibrotic changes involving the atrioventricular node, and myocyte hypertrophy in morbidly obese subjects [65, 66]. Studies have also found that obesity and morbid obesity is associated with alterations of the heart rate, pulse rate interval, QRS duration, QTc interval, P-, QRS-, T-wave axes and low QRS voltage [67, 68], which have been associated with increased risk of sudden cardiac death [69–72], and that weight loss by bariatric surgery reduced some of these alterations [73]. In one study the multivariable-adjusted hazard ratio for a BMI of ≥ 35 versus 21.0–22.9 was 2.18 (95% CI 1.44–3.28) and when further adjusted for potential intermediates, including high cholesterol, hypertension, diabetes, angina, and heart failure it was attenuated to 1.72 (95% CI 1.13–2.60), suggesting that part of the association might be mediated by these traditional risk factors, but that other risk factors (e.g. left ventricular hypertrophy, resting heart rate and others) also could contribute to the increased risk.

Our meta-analysis has several strengths including the prospective design of the included studies which avoids recall bias and reduces the possibility for selection bias, and with > 3300 cases and > 406,000 participants there was sufficient statistical power to detect moderate associations. Additional strengths include the detailed dose–response analyses which clarified the shape of the dose–response relationship and the robustness of the findings in multiple subgroup analyses as well as the high study quality of the included studies.

The findings have important clinical and public health implications because of the increasing prevalence of overweight and obesity worldwide [1], thus if current trends continue unabated it might contribute to an increased rates of sudden cardiac death.

In conclusion, this meta-analysis suggest an increased risk of sudden cardiac death with increasing BMI and waist-to-hip ratio, however, further studies with adjustment for confounding factors and with stratified analyses by smoking status are needed of waist circumference, weight changes and adiposity at younger ages.

## Electronic supplementary material

Below is the link to the electronic supplementary material.

Supplementary material 1 (DOCX 121 kb)
