# Supplementary material for: Body mass index, abdominal fatness, and the risk of sudden cardiac death: a systematic review and dose–response meta-analysis of prospective studies
Source: Eur J Epidemiol. 2018 Feb 7;33(8):711–22. doi: 10.1007/s10654-017-0353-9 (PMC6061127; doi:10.1007/s10654-017-0353-9)
Supplement: Supplementary file 1 — Supplementary material 1 (DOCX 121 kb) [file 10654_2017_353_MOESM1_ESM.docx]

Supplementary Table 1. Search terms used in the PubMed search

| 1 | body mass index[MeSH] |
| --- | --- |
| 2 | "body mass index" |
| 3 | BMI |
| 4 | overweight[MeSH] |
| 5 | overweight |
| 6 | obesity[MeSH] |
| 7 | obesity |
| 8 | anthropometry[MeSH] |
| 9 | anthropometry |
| 10 | fatness |
| 11 | "body fatness" |
| 12 | "abdominal fatness" |
| 13 | abdominal obesity[MeSH] |
| 14 | abdominal obesity |
| 15 | "waist circumference" |
| 16 | "hip circumference" |
| 17 | waist-to-hip ratio[MeSH] |
| 18 | "waist-to-hip ratio" |
| 19 | adiposity[MeSH] |
| 20 | adiposity |
| 21 | weight gain[MeSH] |
| 22 | "weight gain" |
| 23 | weight change[MeSH] |
| 24 | "weight change" |
| 25 | weight loss[MeSH] |
| 26 | "weight loss" |
| 27 | sudden cardiac death[MeSH] |
| 28 | "sudden cardiac death" |
| 29 | cardiac arrest[MeSH] |
| 30 | "cardiac arrest" |
| 31 | "case-control" |
| 32 | cohort |
| 33 | prospective |
| 34 | longitudinal |
| 35 | retrospective |
| 36 | "follow-up" |
| 37 | "cross-sectional" |
| 38 | "hazard ratio" |
| 39 | "hazard ratios" |
| 40 | "relative risk" |
| 41 | "relative risks" |
| 42 | "incidence rate ratio" |
| 43 | "incidence rate ratios" |
| 44 | "odds ratio" |
| 45 | "odds ratios" |
| 46 | incidence |
| 47 | 1 OR 2 OR 3 OR 4 OR 5 OR 6 OR 7 OR 8 OR 9 OR 10 OR 11 OR 12 OR 13 OR 14 OR 15 OR 16 OR 17 OR 18 OR 19 OR 20 OR 21 OR 22 OR 23 OR 24 OR 25 OR 26 |
| 48 | 27 OR 28 OR 29 OR 30 |
| 49 | 31 OR 32 OR 33 OR 34 OR 35 OR 36 OR 37 OR 38 OR 39 OR 40 OR 41 OR 42 OR 43 OR 44 OR 45 OR 46 |
| 50 | 47 AND 48 AND 49 |

Supplementary Table 2. Search terms used in the Embase search

| 1 | body mass index |
| --- | --- |
| 2 | body mass index/ |
| 3 | BMI |
| 4 | BMI/ |
| 5 | overweight |
| 6 | overweight/ |
| 7 | obesity |
| 8 | obesity/ |
| 9 | anthropometry |
| 10 | anthropometry/ |
| 11 | fatness |
| 12 | Fatness/ |
| 13 | body fatness |
| 14 | body fatness/ |
| 15 | abdominal fatness |
| 16 | abdominal fatness/ |
| 17 | abdominal obesity |
| 18 | abdominal obesity/ |
| 19 | waist circumference |
| 20 | waist circumference/ |
| 21 | hip circumference |
| 22 | hip circumference/ |
| 23 | waist-to-hip ratio |
| 24 | waist-to-hip ratio/ |
| 25 | adiposity |
| 26 | adiposity/ |
| 27 | weight gain |
| 28 | weight gain/ |
| 29 | weight change |
| 30 | weight change/ |
| 31 | weight loss |
| 32 | weight loss/ |
| 33 | sudden cardiac death |
| 34 | sudden cardiac death/ |
| 35 | cardiac arrest |
| 36 | cardiac arrest/ |
| 37 | case-control |
| 38 | cohort |
| 39 | prospective |
| 40 | longitudinal |
| 41 | retrospective |
| 42 | follow-up |
| 43 | cross-sectional |
| 44 | hazard ratio |
| 45 | hazard ratios |
| 46 | relative risk |
| 47 | relative risks |
| 47 | incidence rate ratio |
| 48 | incidence rate ratios |
| 49 | odds ratio |
| 50 | odds ratios |
| 51 | incidence |
| 52 | 1 OR 2 OR 3 OR 4 OR 5 OR 6 OR 7 OR 8 OR 9 OR 10 OR 11 OR 12 OR 13 OR 14 OR 15 OR 16 OR 17 OR 18 OR 19 OR 20 OR 21 OR 22 OR 23 OR 24 OR 25 OR 26 OR 27 OR 28 OR 29 OR 30 OR 31 OR 32 |
| 53 | 33 OR 34 OR 35 OR 36 |
| 54 | 37 OR 38 OR 39 OR 40 OR 41 OR 42 OR 43 OR 44 OR 45 OR 46 OR 47 OR 48 OR 49 OR 50 OR 51 |
| 55 | 52 AND 53 AND 54 |

Supplementary Table 3. List of excluded studies and reason for exclusion

| **Exclusion reason** | **Reference number** |
| --- | --- |
| Abstract only publication | (1-8) |
| Case-control study | (9-12) |
| Case only study | (13;14) |
| Comparison group was MI survivors, not the total non-case population | (15) |
| Duplicates | (16-18) |
| Letter, comment | (19-22) |
| Metabolic syndrome | (23) |
| No confidence intervals | (24) |
| No risk estimates | (25;26) |
| Not relevant data | (27) |
| Not relevant exposure | (28-41) |
| Not relevant outcome | (42-50) |
| Outcome was cardiac arrest, not sudden cardiac death | (51-53) |
| Patient populations | (54-58) |
| Review | (59-64) |
| Survival after cardiac arrest | (65;66) |

Reference List

1. Shadman R, Poole JE, Mozaffarian D et al. Predicting the proportional risk of sudden cardiac death in a multicenter heart failure cohort. Circulation Conference: American Heart Association's Scientific Sessions 2011;124:A17819.

2. Adabag S, Lopez F, Alonso A et al. Risk of sudden cardiac death in obese individuals: The atherosclerosis risk in communities (ARIC) study. Heart Rhythm Conference: 33rd Annual Scientific Sessions of the Heart Rhythm Society, Heart Rhythm 2012;9:S111-S112.

3. Chiuve SE, Sun Q, Sandhu RK, Tedrow U, Manson JE, Albert CM. Adiposity over the life-course and risk of sudden cardiac death in women. Circulation Conference: American Heart Association 2013;128:A17400.

4. Tung P, Van DM, Rexrode KM, Gaziano JM, Albert CM. Abdominal and total adiposity and sudden cardiac death risk in men. Circulation Conference: American Heart Association 2013;128:A16078.

5. Adabag S, Lopez FL, Alonso A et al. Abdominal obesity is an independent risk factor for sudden cardiac death in non-smoking individuals: The atherosclerosis risk in communities study. Circulation Conference: American Heart Association 2013;128:A15872.

6. Colling RT, Beavers SPR, Gallagher PJ. Sudden cardiac death and body mass index. Journal of Pathology Conference: 7th Joint Meeting of the British Division of the International Academy of Pathology and the Pathological Society of Great Britain and Ireland Edinburgh United Kingdom Conference Publication 2013;231:S28.

7. Ohlsson MA, Kennedy LM, Juhlin T, Melander O. Differences in risk factors and outcome of cardiac arrest in southern Sweden depending on cardiac or non-cardiac origin. European Heart Journal Conference: European Society of Cardiology, ESC Congress 2015;P1395.

8. Finocchiaro G, Dhutia H, Papadakis M et al. Obesity and sudden Death: Data from a large pathology registry. European Journal of Preventive Cardiology Conference: EuroPRevent 2017;102:A67.

9. Kahn HS, Simoes EJ, Koponen M, Hanzlick R. The abdominal diameter index and sudden coronary death in men. Am J Cardiol 1996;78:961-4.

10. Wisten A, Messner T. Young Swedish patients with sudden cardiac death have a lifestyle very similar to a control population. Scand Cardiovasc J 2005;39:137-42.

11. Filippi A, Sessa J, Mazzaglia G et al. Out of hospital sudden cardiac death in Italy: A population-based case-control study. Journal of Cardiovascular Medicine 9 (6) (pp 595-600), 2008;9:595-600.

12. Yang J, Teehan D, Farioli A, Baur DM, Smith D, Kales SN. Sudden cardiac death among firefighters </=45 years of age in the United States. Am J Cardiol 2013;112:1962-7.

13. Noheria A, Teodorescu C, Uy-Evanado A et al. Distinctive profile of sudden cardiac arrest in middle-aged vs. older adults: a community-based study. Int J Cardiol 2013;168:3495-9.

14. Srivatsa UN, Swaminathan K, Sithy Athiya MK, Amsterdam E, Shantaraman K. Sudden cardiac death in South India: Incidence, risk factors and pathology. Indian Pacing Electrophysiol J 2016;16:121-5.

15. Andersson J, Wennberg P, Lundblad D, Escher SA, Jansson JH. Diabetes mellitus, high BMI and low education level predict sudden cardiac death within 24 hours of incident myocardial infarction. Eur J Prev Cardiol 2016;23:1814-20.

16. Chiuve SE, Fung TT, Rexrode KM et al. Adherence to a low-risk, healthy lifestyle and risk of sudden cardiac death among women. JAMA 2011;306:62-9.

17. Albert CM, Chae CU, Grodstein F et al. Prospective study of sudden cardiac death among women in the United States. Circulation 2003;107:2096-101.

18. Jouven X, Zureik M, Desnos M, Guerot C, Ducimetiere P. Resting heart rate as a predictive risk factor for sudden death in middle-aged men. Cardiovasc Res 2001;50:373-8.

19. Morrow JP. High-fat diet, obesity and sudden cardiac death. Acta Physiol (Oxf) 2014;211:13-6.

20. Jae SY, Kurl S, Laukkanen JA, Franklin BA. Physical activity and cardiorespiratory fitness as underappreciated modulators of obesity-related risk of sudden cardiac death. Heart 2015;101:822.

21. Chirumbolo S. Heart adipose tissue and sudden cardiac death in middle-aged men. International Journal of Cardiology 214 (pp 362-363), 2016;214:362-3.

22. Kawada T. Metabolic syndrome and sudden cardiac death: Validity on risk assessment. International Journal of Cardiology 2016;212:109.

23. Empana JP, Duciemetiere P, Balkau B, Jouven X. Contribution of the metabolic syndrome to sudden death risk in asymptomatic men: the Paris Prospective Study I. Eur Heart J 2007;28:1149-54.

24. Cupples LA, Gagnon DR, Kannel WB. Long- and short-term risk of sudden coronary death. Circulation 1992;85:I11-I18.

25. Eijgelsheim M, Newton-Cheh C, Aarnoudse AL et al. Genetic variation in NOS1AP is associated with sudden cardiac death: evidence from the Rotterdam Study. Hum Mol Genet 2009;18:4213-8.

26. Maruyama M, Ohira T, Imano H et al. Trends in sudden cardiac death and its risk factors in Japan from 1981 to 2005: The Circulatory Risk in Communities Study (CIRCS). BMJ Open 2012;2:e000573.

27. Duflou J, Virmani R, Rabin I, Burke A, Farb A, Smialek J. Sudden death as a result of heart disease in morbid obesity. Am Heart J 1995;130:306-13.

28. Burke AP, Farb A, Pestaner J et al. Traditional risk factors and the incidence of sudden coronary death with and without coronary thrombosis in blacks. Circulation 2002;105:419-24.

29. Maradit-Kremers H, Crowson CS, Nicola PJ et al. Increased unrecognized coronary heart disease and sudden deaths in rheumatoid arthritis: A population-based cohort study. Arthritis and Rheumatism 52 (2) (pp 402-411), 2005;52:402-11.

30. Wennberg P, Eliasson M, Hallmans G, Johansson L, Boman K, Jansson J-H. The risk of myocardial infarction and sudden cardiac death amongst snuff users with or without a previous history of smoking. Journal of Internal Medicine 262 (3) (pp 360-367), 2007;262:360-7.

31. Whang W, Kubzansky LD, Kawachi I et al. Depression and Risk of Sudden Cardiac Death and Coronary Heart Disease in Women. Results From the Nurses' Health Study. Journal of the American College of Cardiology 53 (11) (pp 950-958), 2009;53:950-8.

32. Laukkanen JA, Mkikallio TH, Rauramaa R, Kiviniemi V, Ronkainen K, Kurl S. Cardiorespiratory fitness is related to the risk of sudden cardiac death: A population-based follow-up study. Journal of the American College of Cardiology 56 (18) (pp 1476-1483), 2010;56:1476-83.

33. Barker DJ, Larsen G, Osmond C, Thornburg KL, Kajantie E, Eriksson JG. The placental origins of sudden cardiac death. Int J Epidemiol 2012;41:1394-9.

34. Okin PM, Kjeldsen SE, Julius S, Dahlof B, Devereux RB. Racial differences in sudden cardiac death among hypertensive patients during antihypertensive therapy: the LIFE study. Heart Rhythm 2012;9:531-7.

35. Laukkanen JA, Jennings JR, Kauhanen J, Makikallio TH, Ronkainen K, Kurl S. Relation of systemic blood pressure to sudden cardiac death. American Journal of Cardiology 110 (3) (pp 378-382), 2012;110:378-82.

36. Havmoeller R, Reinier K, Teodorescu C et al. Elevated plasma free fatty acids are associated with sudden death: A prospective community-based evaluation at the time of cardiac arrest. Heart Rhythm 11 (4) (pp 691-696), 2014;11:691-6.

37. Jimenez-Pavon D, Artero EG, Lee DC et al. Cardiorespiratory Fitness and Risk of Sudden Cardiac Death in Men and Women in the United States: A Prospective Evaluation From the Aerobics Center Longitudinal Study. Mayo Clin Proc 2016;91:849-57.

38. Smallman DP, Webber BJ, Mazuchowski EL, Scher AI, Jones SO, Cantrell JA. Sudden cardiac death associated with physical exertion in the US military, 2005-2010. Br J Sports Med 2016;50:118-23.

39. Kurl S, Laaksonen DE, Jae SY et al. Metabolic syndrome and the risk of sudden cardiac death in middle-aged men. Int J Cardiol 2016;203:792-7.

40. Kunutsor SK, Khan H, Nyyssonen K, Laukkanen JA. Lipoprotein(a) and risk of sudden cardiac death in middle-aged Finnish men: A new prospective cohort study. International Journal of Cardiology 2016;220:718-25.

41. Kunutsor SK, Zaccardi F, Karppi J, Kurl S, Laukkanen JA. Is High Serum LDL/HDL Cholesterol Ratio an Emerging Risk Factor for Sudden Cardiac Death? Findings from the KIHD Study. J Atheroscler Thromb 2017;24:600-8.

42. Aronow WS, Herzig AH, Etienne F, D'Alba P, Ronquillo J. 41-month follow-up of risk factors correlated with new coronary events in 708 elderly patients. J Am Geriatr Soc 1989;37:501-6.

43. Walker M, Wannamethee G, Whincup PH, Shaper AG. Weight change and risk of heart attack in middle-aged British men. Int J Epidemiol 1995;24:694-703.

44. Rich-Edwards JW, Kleinman K, Michels KB et al. Longitudinal study of birth weight and adult body mass index in predicting risk of coronary heart disease and stroke in women. BMJ 2005;330:1115.

45. Vukmir RB. Association of obesity with worsened prehospital cardiac arrest. Journal of Nutritional and Environmental Medicine 2005;15:1-8.

46. Saito Y, Kita T, Mabuchi H et al. Obesity as a risk factor for coronary events in Japanese patients with hypercholesterolemia on low-dose simvastatin therapy. J Atheroscler Thromb 2010;17:270-7.

47. Al-Refaie WB, Parsons HM, Henderson WG et al. Body mass index and major cancer surgery outcomes: Lack of association or need for alternative measurements of obesity? Annals of Surgical Oncology 2010;17:2264-73.

48. Talaei M, Sadeghi M, Marshall T et al. Impact of metabolic syndrome on ischemic heart disease - a prospective cohort study in an Iranian adult population: Isfahan Cohort Study. Nutr Metab Cardiovasc Dis 2012;22:434-41.

49. Beckett V, Knight M, Sharpe P. The CAPS Study: Incidence, management and outcomes of cardiac arrest in pregnancy in the UK: A prospective, descriptive study. BJOG: An International Journal of Obstetrics and Gynaecology (no pagination), 2017;124:1374-81.

50. Puddu PE, Terradura VO, Mancini M, Zanchetti A, Menotti A. Typical and atypical coronary heart disease deaths and their different relationships with risk factors. The Gubbio residential cohort study. Int J Cardiol 2014;173:300-4.

51. Ohlsson MA, Kennedy LMA, Juhlin T, Melander O. Midlife risk factor exposure and incidence of cardiac arrest depending on cardiac or non-cardiac origin. Int J Cardiol 2017;240:398-402.

52. Thorgeirsson G, Thorgeirsson G, Sigvaldason H, Witteman J. Risk factors for out-of-hospital cardiac arrest: the Reykjavik Study. Eur Heart J 2005;26:1499-505.

53. Galatianou I, Karlis G, Apostolopoulos A et al. Body mass index and outcome of out-of-hospital cardiac arrest patients not treated by targeted temperature management. Am J Emerg Med 2017;35:1247-51.

54. Drenick EJ, Fisler JS. Sudden cardiac arrest in morbidly obese surgical patients unexplained after autopsy. Am J Surg 1988;155:720-6.

55. Shadman R, Poole JE, Dardas TF et al. A novel method to predict the proportional risk of sudden cardiac death in heart failure: Derivation of the Seattle Proportional Risk Model. Heart Rhythm 2015;12:2069-77.

56. Choy B, Hansen E, Moss AJ, McNitt S, Zareba W, Goldenberg I. Relation of Body Mass Index to Sudden Cardiac Death and the Benefit of Implantable Cardioverter-Defibrillator in Patients With Left Ventricular Dysfunction After Healing of Myocardial Infarction. American Journal of Cardiology 2010;105:581-6.

57. Benchimol D, Dubroca B, Bernard V et al. Short- and long-term risk factors for sudden death in patients with stable angina. Int J Cardiol 2000;76:147-56.

58. Gastelurrutia P, Pascual-Figal D, Vazquez R et al. Obesity paradox and risk of sudden death in heart failure results from the MUerte Subita en Insuficiencia cardiaca (MUSIC) study. Am Heart J 2011;161:158-64.

59. Contaldo F, Pasanisi F, Finelli C, de SG. Obesity, heart failure and sudden death. Nutr Metab Cardiovasc Dis 2002;12:190-7.

60. Chugh SS, Reinier K, Teodorescu C et al. Epidemiology of sudden cardiac death: clinical and research implications. Prog Cardiovasc Dis 2008;51:213-28.

61. Anand RG, Peters RW, Donahue TP. Obesity and dysrhythmias. J Cardiometab Syndr 2008;3:149-54.

62. Lavie CJ, Milani RV, Ventura HO. Obesity and cardiovascular disease: risk factor, paradox, and impact of weight loss. J Am Coll Cardiol 2009;53:1925-32.

63. Chrostowska M, Szyndler A, Hoffmann M, Narkiewicz K. Impact of obesity on cardiovascular health. Best Pract Res Clin Endocrinol Metab 2013;27:147-56.

64. Plourde B, Sarrazin JF, Nault I, Poirier P. Sudden cardiac death and obesity. Expert Rev Cardiovasc Ther 2014;12:1099-110.

65. Gupta T, Kolte D, Mohananey D et al. Relation of Obesity to Survival After In-Hospital Cardiac Arrest. Am J Cardiol 2016;118:662-7.

66. Jain R, Nallamothu BK, Chan PS. Body mass index and survival after in-hospital cardiac arrest. Circ Cardiovasc Qual Outcomes 2010;3:490-7.

Supplementary Table 4: Prospective studies of adiposity and sudden cardiac death

| Author, publication year, country/ region | Study name | Recruitment and follow-up period | Study size, gender, age, number of cases | Assessment of weight and height | Exposure by subgroup | Description of quantiles of categories | RR (95% CI) | Adjustment for confounders |
| --- | --- | --- | --- | --- | --- | --- | --- | --- |
| Wannamethee G et al, 1995, United Kingdom | British Regional Heart Study | 1978-1980 - NA, 8 years follow-up | 7735 men, age 40-59 years: 117 sudden cardiac deaths | Measured | BMI | <22.0  22.0-23.9  24.0-25.9  26.0-27.9  ≥28.0 | 1.0  0.8 (0.4-1.8)  1.3 (0.6-2.6)  1.3 (0.6-2.6)  1.6 (0.93-3.2) | Age |
| Jouven X et al, 1999, France | Paris Prospective Study 1 | 1967-1972 - 1994, 23 years follow-up | 7746 men, age 43-52 years: 118 sudden deaths | Measured | BMI | Per 3.3 units | 1.21 (1.03-1.87) | Age, tobacco, DM, heart rate, SBP, cholesterol, TG, parental MI, parental sudden death |
| Kataoka M et al, 2004, Japan | Hiroshima Atomic Bomb Casualty Council Health Management and Promotion Center | 1983-2001, 5.2 years follow-up | 8917 men and women, age 35-69 years: 56 sudden cardiac deaths | NA | BMI | Per 1 unit | 0.98 (0.90-1.07) | Age, sex, ischemic ECG change, CV_R-R_, SBP, total cholesterol, triglycerides, diabetes, smoking |
| Chei CL et al, 2008, Japan | Japan Public Health-Center Based Prospective Study | 1990-1993 - 2001, 9.7 years follow-up | 43235 men and 47444 women, age 40-69 years: 65/24 sudden cardiac deaths | Self-reported (validated) | BMI, men  BMI, women | 14.0-18.9  19.0-20.9  21.0-22.9  23.0-24.9  25.0-26.9  27.0-29.9  30.0-39.9  14.0-18.9  19.0-20.9  21.0-22.9  23.0-24.9  25.0-26.9  27.0-29.9  30.0-39.9 | 2.4 (1.0-5.8)  1.1 (0.5-2.4)  0.9 (0.5-1.8)  1.0  0.8 (0.3-1.7)  0.4 (0.1-1.5)  1.4 (0.3-6.1)  3.5 (1.0-12.7)  0.6 (0.1-3.1)  1.2 (0.4-3.6)  1.0  1.2 (0.4-3.8)  -  - | Age, smoking status, cigarettes per day, alcohol, hypertension, diabetes, leisure-time sports, green vegetables, fish, public health centre |
| Laukkanen JA et al, 2010, Finland | Kuopio Ischemic Heart Disease Risk Factor Cohort | 1984-1989 - 2005, 17.6 years follow-up | 2368 men, age 42-60 years: 146 sudden cardiac deaths | Measured | Waist-to-hip ratio | Per 0.06 units | 1.19 (0.35-4.04) | Age, alcohol, cigarette smoking, LDL cholesterol, HDL cholesterol, hsCRP, type 2 diabetes, SBP, CHD, FH - CHD, maximal heart rate, ischemic ST-segment changes during exercise, cardiorespiratory fitness, Harrell C-index |
| Ohira T et al, 2012, Japan | Circulatory Risk in Communities Study | 1975-2005, ~3.5 years follow-up | 26870 men and women, nested case-control study:  239 sudden cardiac deaths  717 controls  Age 30-84 years | Measured | BMI | <18.5  18.5-24.9  25.0-29.9  ≥30.0 | 1.36 (0.80-2.32)  1.00  0.99 (0.70-1.40)  1.52 (0.75-3.06) | Age, sex, examination year, follow-up time, community, hypertension, diabetes, hyperlipidemia, excess ethanol intake, current smoking, heart rate, atrial fibrillation, SVPC/VPC, major ST-T abnormalities, minor ST-T abnormalities, prolonged PQ duration, wide QRS, left high amplitude R wave, abnormal Q wave |
| Bertoia ML et al, 2012, USA | Women’s Health Initiative | 1993-1998 - 2009, 10.8 years | 161808 women, age 50-79 years: 418 sudden cardiac death cases | Measured | BMI, all  WHR  BMI, without prior CHD  WHR | 18.5-<25  25.0-<30  ≥30.0  ≤0.76  0.77-0.80  0.81-0.86  ≥0.87  18.5-<25  25.0-<30  ≥30.0  ≤0.76  0.77-0.80  0.81-0.86  ≥0.87 | 1.00  1.00 (0.75-1.33)  1.15 (0.86-1.56)  1.00  0.90 (0.60-1.35)  1.23 (0.85-1.78)  1.73 (1.20-2.48)  1.00  1.06 (0.73-1.52)  1.21 (0.82-1.78)  1.00  1.05 (0.63-1.75)  1.68 (1.05-2.70)  2.02 (1.26-3.24) | Age, race, family income, smoking, resting pulse, white blood cell count, CHD, MI, heart failure, atrial fibrillation, diabetes mellitus, carotid artery disease, hypertension, mutual adjustment between BMI and WHR |
| Lahtinen AM et al, 2012, Finland | FINRISK 1992 | 1992-2008, ~16 years follow-up | 5345 men and women, mean age 44.3 years: 129 sudden cardiac deaths | Measured | BMI | Per 1 unit | 1.05 (1.02-1.09) | Age, sex, geographic region, HDL/TC ratio, SBP, smoking, diabetes, physical activity, prevalent CHD, QT-prolonging drug, digoxin |
| Lahtinen AM et al, 2012, Finland | FINRISK 1997 | 1997-2008, ~11 years follow-up | 7672 men and women, mean age 48.4 years: 178 sudden cardiac deaths | Measured | BMI | Per 1 unit | 1.01 (0.97-1.04) | Age, sex, geographic region, HDL/TC ratio, SBP, smoking, diabetes, physical activity, prevalent CHD, QT-prolonging drug, digoxin |
| Lahtinen AM et al, 2012, Finland | FINRISK 2002 | 2002-2008, ~6 years follow-up | 8212 men and women, mean age 48.0 years: 75 sudden cardiac deaths | Measured | BMI | Per 1 unit | 1.04 (0.99-1.09) | Age, sex, geographic region, HDL/TC ratio, SBP, smoking, diabetes, physical activity, prevalent CHD, QT-prolonging drug, digoxin |
| Lahtinen AM et al, 2012, Finland | Health 2000 | 2000-2008, ~8 years follow-up | 6400 men and women, mean age 53.0 years: 112 sudden cardiac deaths | Measured | BMI | Per 1 unit | 0.97 (0.92-1.01) | Age, sex, geographic region, HDL/TC ratio, SBP, smoking, diabetes, physical activity, prevalent CHD, QT-prolonging drug, digoxin |
| Karppi J et al, 2013, Finland | Kuopio Ischemic Heart Disease Risk Factor Cohort | 1984-1989 - 2008, 15.9 years follow-up | 1031 men, age 46-65 years: 59 sudden cardiac deaths | Measured | Waist circumference | Per 1 cm | 1.01 (0.98-1.03) | Age, SBP, diabetes, current smoking, alcohol, education, prevalent CHD, CHF, MI, hypertension, serum beta-carotene, LDL cholesterol, triglycerides, hs-CRP |
| Laukkanen JA et al, 2013, Finland | Kuopio Ischemic Heart Disease Risk Factor Cohort | 1984-1989 - 2008, 18.8 years follow-up | 2641 men, age 42-60 years: 190 sudden cardiac deaths | Measured | BMI | Per 5 units | 1.38 (1.15-1.67) | Age, fasting plasma glucose, cigarette smoking, prevalent CHD, SBP, LDL cholesterol, FH - CHD, alcohol |
| Adabag S et al, 2015, USA | Atherosclerosis Risk in Communities Study | 1987-1989 - 2011-2013, 12.6 years follow-up | 14941 men and women, age 45-64 years: 253 sudden cardiac deaths | Measured | BMI, non-smokers  BMI, current smokers  Waist circumference, non-smokers  Waist circumference, current smokers  WHR, non-smokers  WHR, current smokers  BMI, non-smokers  BMI, current smokers  Waist circumference, non-smokers  Waist circumference, current smokers  WHR, non-smokers  WHR, current smokers | <18.5  18.5-24.9  25.0-29.9  30.0-34.9  ≥35.0  <18.5  18.5-24.9  25.0-29.9  30.0-34.9  ≥35.0  <88/<102 cm  88-95/102-105  96-106/106-111  107-168/112-178  <88/<102 cm  88-95/102-105  96-106/106-111  107-168/112-178  <0.80/<0.95  0.80-0.87/0.95-0.974  0.88-0.94/0.975-1.009  0.95-1.29/1.01-1.39  <0.80/<0.95  0.80-0.87/0.95-0.974  0.88-0.94/0.975-1.009  0.95-1.29/1.01-1.39  <18.5  18.5-24.9  25.0-29.9  30.0-34.9  ≥35.0  <18.5  18.5-24.9  25.0-29.9  30.0-34.9  ≥35.0  <88/<102 cm  88-95/102-105  96-106/106-111  107-168/112-178  <88/<102 cm  88-95/102-105  96-106/106-111  107-168/112-178  <0.80/<0.95  0.80-0.87/0.95-0.974  0.88-0.94/0.975-1.009  0.95-1.29/1.01-1.39  <0.80/<0.95  0.80-0.87/0.95-0.974  0.88-0.94/0.975-1.009  0.95-1.29/1.01-1.39 | -  1.00  1.73 (1.06-2.84)  1.94 (1.12-3.33)  3.36 (1.85-6.07)  1.39 (0.43-4.50)  1.00  0.86 (0.55-1.33)  0.94 (0.53-1.65)  0.34 (0.08-1.41)  1.00  1.22 (0.74-2.01)  1.25 (0.76-2.03)  2.35 (1.56-3.54)  1.00  1.25 (0.73-2.13)  0.99 (0.55-1.77)  0.75 (0.38-1.49)  1.00  1.15 (0.63-2.11)  2.29 (1.39-3.77)  3.54 (2.15-5.81)  1.00  0.96 (0.50-1.85)  1.96 (1.15-3.33)  1.48 (0.83-2.63)  -  1.00  1.27 (0.77-2.10)  1.06 (0.60-1.87)  1.46 (0.78-2.76)  1.82 (0.55-6.03)  1.00  0.66 (0.42-1.04)  0.60 (0.33-1.09)  0.16 (0.04-0.69)  1.00  0.89 (0.54-1.48)  0.89 (0.54-1.47)  1.27 (0.81-2.00)  1.00  1.05 (0.60-1.83)  0.65 (0.35-1.20)  0.42 (0.20-0.85)  1.00  1.00 (0.54-1.84)  1.47 (0.88-2.46)  2.03 (1.19-3.46)  1.00  0.71 (0.36-1.37)  1.27 (0.73-2.21)  0.76 (0.41-1.42) | Age, sex, race, field centre, education  +SBP, antihypertensive medication use, diabetes, LDL cholesterol, HDL cholesterol, triglycerides, prevalent CHD, prevalent HF, resting heart rate on ECG, LVH at baseline ECG |
| Chiuve SE et al, 2015, USA | Nurses' Health Study | 1980-2012, 32 years follow-up | 72484 women, age 34-59 years: 445 Sudden cardiac deaths | Self-reported (validated) | BMI, updated every 2 years  BMI, baseline  BMI at age 18 years  Weight change | 18.5-20.9  21.0-22.9  23.0-24.9  25.0-29.9  30.0-34.9  ≥35.0  18.5-20.9  21.0-22.9  23.0-24.9  25.0-29.9  30.0-34.9  ≥35.0  18.5-20.9  21.0-22.9  23.0-24.9  25.0-29.9  30.0-34.9  ≥35.0  - >5 kg  Stable  + 5-9.9 kg  + 10-19.9  + ≥20 | 1.50 (0.99-2.28)  1.00  0.96 (0.65-1.42)  1.46 (1.05-2.04)  1.46 (1.00-2.13)  2.18 (1.44-3.28)  0.94 (0.64-1.40)  1.00  0.99 (0.71-1.38)  1.69 (1.26-2.25)  2.42 (1.72-3.41)  3.25 (2.16-4.90)  0.88 (0.70-1.12)  1.00  1.33 (1.00-1.76)  1.33 (0.96-1.84)  2.09 (1.27-3.43)  3.92 (1.67-9.18)  0.79 (0.50-.23)  1.00  1.14 (0.86-1.52)  1.41 (1.06-1.88)  2.08 (1.53-2.83) | Age, calendar time, smoking, physical activity, alcohol, total energy intake, FH - MI, current use of hormone therapy, menopausal status, aspirin use, multivitamins, SFA, n-3 PUFA, n-6 PUFA, magnesium, diabetes, hypercholesterolemia, hypertension |
| Eranti A et al, 2016, Finland | Social Insurance Intitution's Coronary Heart Disease study | 1966-1972 - 2007, ~38 years follow-up | 10543 men and women, age 30-59 years: 769 Sudden cardiac deaths | Measured | BMI | <20  20.0-24.9  25.0-29.9  ≥30.0 | 1.11 (0.70-1.77)  1.00  1.33 (1.13-1.56)  1.79 (1.44-2.23) | Age, sex, smoking status, SBP, diabetes, cholesterol, baseline cardiac disease, any ECG abnormality |

CHD=coronary heart disease, CHF=congestive heart failure, hsCRP=high-sensitive C-reactive protein, DM=diabetes mellitus, ECG=electrocardiography, FH=family history, HDL=high-density lipoprotein, HF=heart failure, LDL=low-density lipoprotein, LVH=left ventricular hypertrophy, MI=myocardial infarction, PUFA=polyunsaturated fatty acids, SBP=systolic blood pressure, SFA=saturated fatty acids, SVPC=supraventricular premature complexes, TC=total cholesterol, TG=triglycerides, VPC=ventricular premature complexes, WHR=waist-to-hip ratio

Supplementary Table 5. Sensitivity analysis excluding one study at a time from the analysis of BMI and sudden cardiac death

------------------------------------------------------------------------------

Study omitted | Estimate [95% Conf. Interval]

-------------------+----------------------------------------------------------

Eranti, 2016 (2016)| 1.1432852 1.0239815 1.2764888

Adabag, 2015 (2015)| 1.1547469 1.0352069 1.2880906

Chiuve, 2015 (2015)| 1.1353823 1.0320773 1.2490276

Laukkanen, 2013 (2013)| 1.1429986 1.0301293 1.2682346

Bertoia, 2012 (2012)| 1.1672924 1.0513009 1.2960813

Lahtinen, 2012, FINRISK 1992 (2012)| 1.1488135 1.0322236 1.2785723

Lahtinen, 2012, FINRISK 1997 (2012)| 1.1727182 1.0578775 1.3000257

Lahtinen, 2012, FINRISK 2002 (2012)| 1.1562998 1.0417734 1.2834166

Lahtinen, 2012, Health 2002 (2012)| 1.1951077 1.0895942 1.3108387

Ohira, 2012 (2012)| 1.1799395 1.0688206 1.3026108

Chei, 2008 (2008) | 1.1927226 1.0910293 1.3038946

Kataoka, 2004 (2004)| 1.1733245 1.0628036 1.2953383

Jouven , 1999 (1999)| 1.1555955 1.0447497 1.2782017

Wannamethee, 1995 (1995)| 1.1484752 1.037833 1.2709129

-------------------+----------------------------------------------------------

Combined | 1.1622135 1.0541251 1.2813852

------------------------------------------------------------------------------

Supplementary Table 6. Table of RRs and 95% CIs from nonlinear dose-response analysis of BMI and sudden cardiac death

|  | Sudden cardiac death |
| --- | --- |
| BMI | RR (95% CI) |
| 16.7 | 1.28 (1.04-1.56) |
| 17.5 | 1.17 (1.02-1.35) |
| 20.0 | 1.00 |
| 22.5 | 0.97 (0.89-1.05) |
| 25.0 | 1.02 (0.91-1.15) |
| 27.5 | 1.14 (1.00-1.30) |
| 30.0 | 1.33 (1.17-1.51) |
| 32.5 | 1.59 (1.41-1.78) |
| 35.0 | 1.94 (1.75-2.15) |
| 37.5 | 2.41 (2.18-2.66) |
| 40.0 | 3.02 (2.71-3.37) |
| p_nonlinearity_ | <0.0001 |

Supplementary Table 7. Table of RRs and 95% CIs from nonlinear dose-response analysis of WHR and sudden cardiac death

|  | Sudden cardiac death |
| --- | --- |
| WHR | RR (95% CI) |
| 0.75 | 1.00 |
| 0.80 | 1.75 (1.43-2.14) |
| 0.85 | 2.71 (1.94-3.78) |
| 0.90 | 3.89 (2.59-5.86) |
| 0.95 | 5.13 (3.33-7.89) |
| 1.00 | 6.11 (4.02-9.28) |
| p_nonlinearity_ | 0.02 |

Supplementary Table 8. Study quality of studies on BMI and sudden cardiac death

| Author, publication year | Represen-tativeness | Selection of non-exposed cohort | Exposure-ascertainment^1^ | Demonstration of outcome not present at start^2^ | Adjustment for one risk factor | Adjustment for any other factor | Assess-ment of outcome | Long enough follow-up | Adequacy of follow-up^3^ | Total score |
| --- | --- | --- | --- | --- | --- | --- | --- | --- | --- | --- |
| Wannamethee, 1995 | 1 | 1 | 1 | 0 | 1 | 0 | 1 | 1 | 1 | 7 |
| Jouven, 1999 | 1 | 1 | 1 | 1 | 1 | 1 | 1 | 1 | 0 | 8 |
| Kataoka, 2004 | 0 | 1 | 0 | 1 | 1 | 1 | 1 | 1 | 1 | 7 |
| Chei, 2008 | 1 | 1 | 1 | 1 | 1 | 1 | 1 | 1 | 0 | 8 |
| Bertoia, 2008 | 1 | 1 | 1 | 0 | 1 | 1 | 1 | 1 | 0 | 7 |
| Lahtinen, 2012, FINRISK 1992 | 1 | 1 | 1 | 0 | 1 | 1 | 1 | 1 | 1 | 8 |
| Lahtinen, 2012, FINRISK 1997 | 1 | 1 | 1 | 0 | 1 | 1 | 1 | 1 | 1 | 8 |
| Lahtinen, 2012, FINRISK 2002 | 1 | 1 | 1 | 0 | 1 | 1 | 1 | 1 | 1 | 8 |
| Lahtinen, 2012, Health 2002 | 1 | 1 | 1 | 0 | 1 | 1 | 1 | 1 | 1 | 8 |
| Ohira, 2012 | 1 | 1 | 0 | 0 | 1 | 1 | 1 | 1 | 0 | 6 |
| Laukkanen, 2013 | 1 | 1 | 1 | 0 | 1 | 1 | 1 | 1 | 1 | 8 |
| Adabag, 2015 | 1 | 1 | 1 | 0 | 1 | 1 | 1 | 1 | 0 | 7 |
| Chiuve, 2015 | 0 | 1 | 1 | 1 | 1 | 1 | 1 | 1 | 1 | 8 |
| Eranti, 2016 | 1 | 1 | 1 | 1 | 1 | 1 | 1 | 1 | 1 | 9 |

^1^ 1 point for validated self-reported or measured weight and height

^2^ 1 point for exclusion of prevalent cardiovascular disease cases at baseline

^3^ 1 point for follow-up of ≥3 years

^4^ 1 point for loss-to-follow-up less than 10%

Supplementary Figure 1. Funnel plot of BMI and sudden cardiac death

Supplementary Figure 2. Nonlinear dose-response analysis of BMI and sudden cardiac death, <10 years follow-up

Supplementary Figure 3. Nonlinear dose-response analysis of BMI and sudden cardiac death, ≥10 years follow-up
